# Reconfigurable optical assembly of nanostructures

**DOI:** 10.1038/ncomms12002

**Published:** 2016-06-23

**Authors:** Yunuen Montelongo, Ali K. Yetisen, Haider Butt, Seok-Hyun Yun

**Affiliations:** 1 Department of Chemistry, Imperial College London, Exhibition Road, South Kensington, London SW7 2AZ, UK; 2 Harvard Medical School and Wellman Center for Photomedicine, Massachusetts General Hospital, 65 Landsdowne Street, Cambridge, Massachusetts 02139, USA; 3 Microengineering and Nanotechnology Laboratory, School of Mechanical Engineering, University of Birmingham, Edgbaston, Birmingham B15 2TT, UK; 4 Harvard-MIT Division of Health Sciences and Technology, Massachusetts Institute of Technology, Cambridge, Massachusetts 02139, USA

## Abstract

Arrangements of nanostructures in well-defined patterns are the basis of photonic crystals, metamaterials and holograms. Furthermore, rewritable optical materials can be achieved by dynamically manipulating nanoassemblies. Here we demonstrate a mechanism to configure plasmonic nanoparticles (NPs) in polymer media using nanosecond laser pulses. The mechanism relies on optical forces produced by the interference of laser beams, which allow NPs to migrate to lower-energy configurations. The resulting NP arrangements are stable without any external energy source, but erasable and rewritable by additional recording pulses. We demonstrate reconfigurable optical elements including multilayer Bragg diffraction gratings, volumetric photonic crystals and lenses, as well as dynamic holograms of three-dimensional virtual objects. We aim to expand the applications of optical forces, which have been mostly restricted to optical tweezers. Holographic assemblies of nanoparticles will allow a new generation of programmable composites for tunable metamaterials, data storage devices, sensors and displays.

## Abstract (web summary)

Reconfigurable materials are of interest for many photonic applications. Here, Montelongo *et al*. demonstrate optical elements such as Bragg diffraction gratings, volumetric photonic crystals, lenses, and holograms in a composite with dispersed nanoparticles which can be recorded and erased.

## Graphical abstract

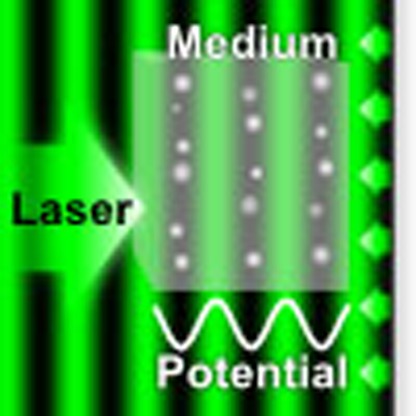

Programmable materials that change their physical properties are a major topic of interest in modern science^1^. Mechanisms to configure nanostructures in three-dimensional (3D) space are essential in nanotechnology, photonics and materials science. Common optical nanofabrication methods rely on light-sensitive materials such as silver halides and photoresists^2^. Highly intense laser pulses can ablate materials with spatial selectivity, altering their physical and optical properties^3^. Various nanopatterning techniques based on photoactive materials or photoablation have been used to produce static photonic crystals^4^, lasers^5^, metamaterials^6^, holograms^7^, storage devices^8^ and sensors^9^. Optically rewritable materials have been demonstrated with photorefractive or photochromic media by changing their refractive indexes locally^10^,^11^,^12^,^13^,^14^. However, they require external energy to maintain the information. Although some dynamic mechanisms have been applied at nanoscale to assemble crystal structures^15^,^16^ and twisted ribbons^17^, the dynamic displacement of 3D nanoassemblies to form well-defined configurations inside solids is not easily accomplished.

Dielectric and metal nanoparticles (NPs) in viscoelastic media have a complex behaviour in the presence of radiation gradients. An optical force (tractor force) results from the momentum transfer associated with the spatially asymmetric light scattering and absorption of a nanostructure^18^. Electromagnetic forces in gradients can push particles towards regions of maximum intensity (positive forces) or minimum intensity (negative forces)^19^. In dielectrics, the force can be positive or negative when the NP has higher or lower refractive indexes than the medium respectively^20^. The phase shift of the scattering dictates the direction of the force. In metal NPs, the phase and intensity of the scattering depends on the surface plasmon resonance produced by the free electron cloud^21^,^22^,^23^. Hence, the direction of the optical force is dictated by different factors including geometry, size and material of the NP, the surrounding medium and the wavelength of the applied field^19^,^24^,^25^. An assembly of NPs embedded in a solid can be reconfigurable with optical forces when the viscoelasticity of the medium permits the migration and the stabilization in a reversible manner. When the optical force passes a threshold, NPs overcome surface adhesion, elastic forces and the static friction induced by the medium.

Here we introduce a strategy based on non-ablative optical pressure to arrange nanostructures inside transparent solids with viscoelastic characteristics. The shape-memory characteristics of some types of polymers provide the necessary plasticity for reversible nanomaterial configuration *in situ*^26^,^27^,^28^. We use optical standing waves to control heat and optical force to arrange NPs in different 3D configurations. In addition to the optical force, thermodynamic (thermophoresis)^29^ and acoustic (acoustophoresis)^30^ forces arise due to mechanical pressure produced by temperature increase in the medium, which pushes NPs towards the minimum light intensity regions (negative force)^31^,^32^,^33^. However, low dissipation of temperature and mechanical pressure are necessary to achieve thermophoresis and acoustophoresis. Although these effects can produce reconfigurability, the system presented in this work is in the optical force regime (Supplementary Notes 1 and 2, and Supplementary Fig. 1). To our knowledge, this optical force-induced mechanism to assemble nanostructures in organized, reversible configurations in solids has not been reported previously. Using this mechanism in the negative force regime, we demonstrate rewritable photonic crystals, optical elements and 3D holograms.

## Results

### Migration of NPs in standing waves

**Figure 1:**
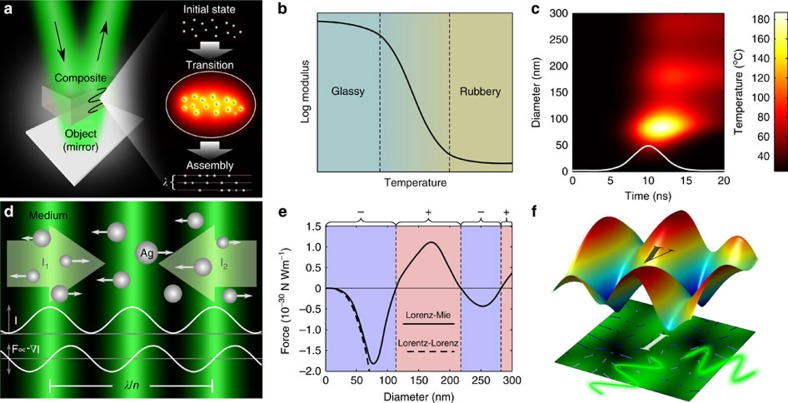
Mechanism of nanostructure reconfiguration in a medium. (**a**) Schematic of the nanoassembly process, which consist of the following: an initial state where NPs are randomly distributed, a transition state where NPs migrate to lower-energy configurations and a final assembly state where NPs are located in new stable positions (Supplementary Movie 1). (**b**) Rheology of a typical thermoplastic (for example, the complex shear modulus (*G*′+*i G*″) of pHEMA changes from 1.4 × 10^9^+*i* 2.0 × 10^7^ Pa in the glassy regime to 2.9 × 10^4^+*i* 2.0 × 10^4^ Pa well above its glass transition temperature (*T*_g_) of 300 °C). (**c**) Time-dependent temperature at the surface of NPs of different diameters from a pulsed laser 532 nm, 5 ns and 20 mJ cm^−2^ (this is equivalent to the temperature produced by two interfering waves of 10 mJ cm^−2^ at the maximum gradient point). (**d**) Dynamics of NP displacement in a standing wave. (**e**) Relative maximum force acting on Ag NPs of different diameters with a standing wave of 532 nm. (**f**) Potential wells produced by the interference of counter-propagating waves.

Metal NPs can absorb optical energy and allow the converted thermal energy to dissipate to their surrounding medium. Hence, the medium behaves as a fluid locally, allowing NPs to be displaced from their original positions. Finally, the composite returns to its original glass state after the displacement (Fig. 1a and Supplementary Movie 1). The analysis of temperature at the NP and medium is necessary to prevent transition of phase. We consider Ag NPs for their high optical scattering and absorption, and poly(2-hydroxyethyl methacrylate) (pHEMA) as the embedding medium for its unique rheological characteristics. The phase transition temperature of Ag NPs is lower than bulk Ag but higher than the degradation temperature of pHEMA, both of which are slightly above 300 °C^34^,^35^. Furthermore, the temperature at the boundary of the NPs dictates the mechanical properties of the surrounding medium. As pHEMA has low heat conduction, the high temperature at the NP–pHEMA boundary allows the pHEMA matrix to behave similar to a viscoelastic rubber. This phenomenon is analogous to ‘the knife in the butter', where the medium changes its stiffness according to the temperature of the metal. Furthermore, this effect is present as long as the heat of the metal diffuses in the pHEMA matrix. We have rationally designed a pHEMA matrix that can reversibly transform from its glass state to its rubber state by increasing the temperature at the NP boundaries (Fig. 1b). A numerical analysis shows the temperature at the boundaries of spherical Ag NPs when they are heated by a Gaussian laser pulse (Fig. 1c). Alternatively, the temperature at the surface *T*_CW_(*t*) in time can be approximated for a continuous irradiance *I*_CW_ as (Supplementary Note 3 and Supplementary Fig. 2):


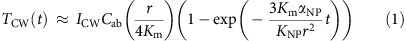


where *C*_ab_ is the absorption efficiency, *K*_NP_ and *α*_NP_ are the thermal conductivity and diffusivity of the NP, respectively, and *K*_m_ is the thermal conductivity of the medium. The optical forces **F**_p_ at the intensity gradient is of the form 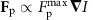. The net optical force of dielectric NPs in standing waves can be positive or negative depending of the refractive indexes. In addition, metal NPs can invert the direction of the force for a wavelength near the surface plasmon resonance. Furthermore, forces in metal NPs can be up to one order of magnitude higher than their dielectric counterpart. An approach to retrieve the force below the first plasmonic resonant mode is with the Lorentz–Lorenz equation^20^:


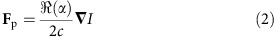


where *c* is the speed of light and 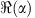 represents the real part of the complex polarizability that can be obtained as:


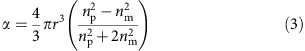


where *n*_p_ and *n*_m_ are complex refractive indexes of a NP and surrounding medium, respectively. This approximation is valid for a Ag NP radius smaller than ∼35 nm. A further effective change in sign can occur for NPs with a size comparable to the period of the standing wave, because the two neighbouring nodes have opposite direction in the force^36^. Depending on the size, NPs settle at the maximum intensity or minimum intensity regions of the interference fringe^37^. A more accurate approximation for metal NPs can be retrieved from the generalized Lorenz–Mie theory (GLMT). In GLMT, the electromagnetic field consisting of incident and scattered field outside the sphere, and the internal field within the sphere, is expanded into a series of multipole terms^19^.

We define arbitrary standing waves with the interference of two coherent beams propagating normal to the plane *z* with an included angle *θ* and intensity *I*_0_. The standing wave created is 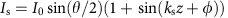, with a wavenumber 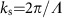, a grating spacing *Λ* and a phase *ϕ*. Furthermore, the phase of the standing wave is controlled with the relative phase difference between the beams, whereas the grating spacing is defined as 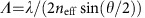, for a given beam wavelength *λ* and a medium effective refractive index of *n*_eff_. Hence, the force exerted by the standing wave of two counter-propagating beams (*θ*=180°) is 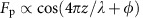. According to the Fourier theorem, an arbitrary intensity profile can be obtained from the superposition of waves^38^. Figure 1d shows the displacement of NPs from bright regions to dark regions in a standing wave produced by a negative force. To retrieve the migration, we applied the generalized Stokes' law for a NP of radius *r* embedded in a complex viscoelastic medium with a shear modulus *G*=2.9 × 10^4^+*i*2.0 × 10^4^ (Supplementary Note 1)^39^:


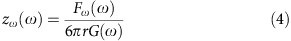


where *z*_ω_(*ω*) is the complex NP displacement and *F*_ω_(*ω*) is the complex force (representing the amplitude and phase of the oscillation). The total migration close to the maximum gradient point produced by the interference of two counter-propagating waves is approximated as:


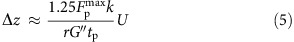


where *k* is the wave number, *U* is the total exposure energy, *t*_p_ is the full width at half maximum pulse size and *F*_p_^max^ is constant that satisfies: 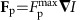, which can be obtained either from the Lorentz–Lorenz or Lorenz–Mie equations.

Figure 1e shows the relative maximum force of Ag NPs considering 532 nm light source for the Lorentz–Lorenz and the Lorenz–Mie equations. The potential energy *V* is proportional to the intensity of the wave *V*∝*I*_s_, as the force correlates with the gradient of the intensity. Figure 1f illustrates the energy potential produced and forces exerted by the interference of four arbitrary waves. The potential wells represent equilibrium positions, because the velocity of NPs is damped by the viscosity of the medium.

### Nanoassembly in composites

**Figure 2:**
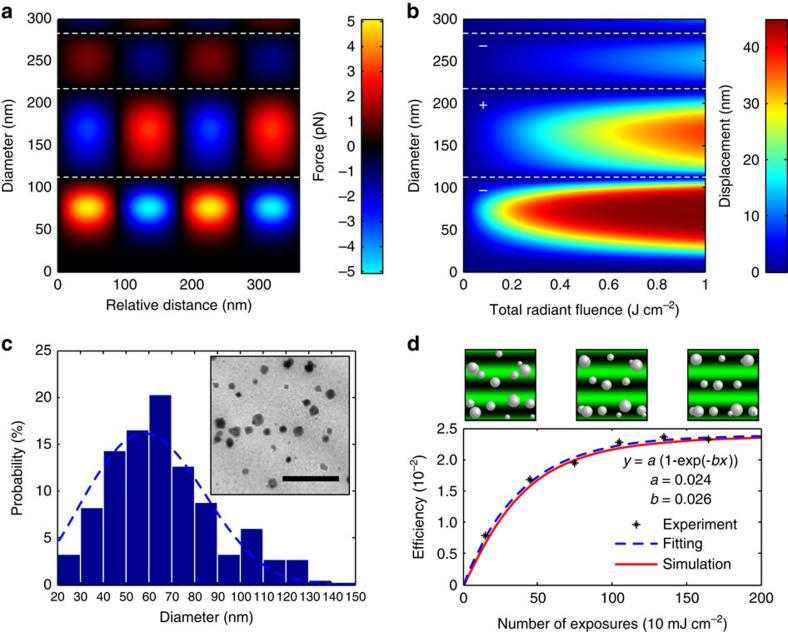
Dynamics of NPs observed from the optical momentum. (**a**) Effect of force at the maximum gradient point produced by a laser pulse (532 nm, 10 mJ cm^−2^, 5 ns) on Ag NP sizes at different locations of the standing wave. (**b**) Magnitude of the displacement from Stokes' law after pulse exposure. (**c**) TEM image showing NP size distribution in the pHEMA matrix (scale bar, 500 nm). (**d**) Comparison between the simulated migration (red line), measured diffraction efficiency (points) and fitted curve (blue dashed line) from a multilayer structure recorded in Denisyuk reflection mode with different exposure numbers.

We calculated the optical force induced by a 532-nm laser pulse of 5 ns and 10 mJ over 1 cm^2^ with the GLMT applied to Ag NPs (Fig. 2a). Figure 2b shows the NP displacement with respect to the total exposure retrieved from Stokes' law (Supplementary Note 1). The maximum force and displacement was observed for NP sizes in the 30–90-nm range. Based on these values, we optimized the fabrication for Ag NPs in the 30–90 nm range embedded in a pHEMA film (∼10 μm in thickness) (see ‘Fabrication of the Recording Media' in Methods). Figure 2c illustrates the optimized size distribution of Ag NPs embedded in the pHEMA matrix. To form the holographic reconfiguration, we used a Nd:YAG (532 nm, 5 ns) pulsed laser. We measured the diffraction efficiency of the recorded multilayer Bragg grating with respect to the incident beam intensity. An interference was produced from two counter-propagating beams in Denisyuk reflection mode with a tilt angle of 5° with respect to the surface plane of the pHEMA matrix^40^. When the radiant fluence was below 1 mJ cm^−2^, no recording effect was observed. However, when the fluence was >20 mJ cm^−2^, the absorption at the NP surface degraded the pHEMA matrix. The optimum fluence was 10 mJ cm^−2^, to maximize the diffraction efficiency. This fluence produced a maximum temperature of 300 °C at the NP surfaces, corresponding to the limit where pHEMA degraded^35^. The NP displacement was increased by repeating the number of pulses without compromising the integrity of the pHEMA matrix. We validated the migration by measuring the diffraction efficiency from the multilayer Bragg grating recorded in Denisyuk reflection mode (see ‘Refractive index measurement' in Methods). Ag NPs were arranged in a slanted 3D structure with a periodicity of ∼*λ*/2. Figure 2d shows the increment in the diffraction efficiency reaching a maximum of 2.4% as the number of pulses increased up to 200 exposures (at 10 Hz). This behaviour is similar to a damped harmonic oscillator, where the dragging force settles the NPs in a steady position. The theoretical curve obtained (red line) agreed with the measured values (points) and the fitted curve (blue dashed line). Based on these parameters, the measured sensitivity of the fabricated composite was 885 mJ cm^−2^ to reach the 90% maximum diffraction efficiency.

**Figure 3:**
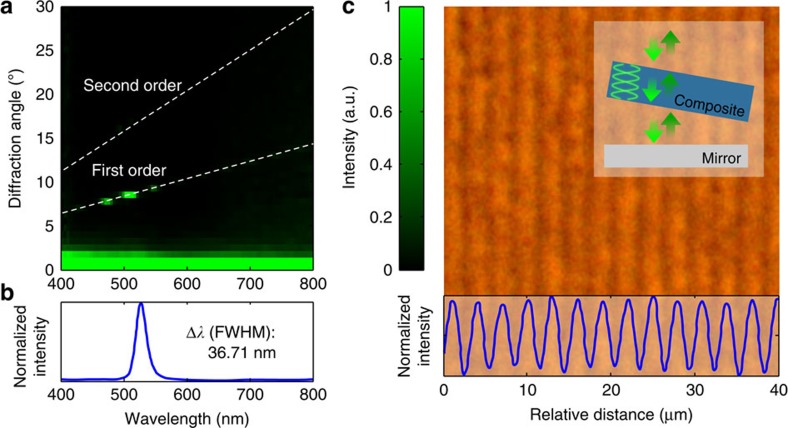
Optical characterization of the multilayer structure. (**a**) Angle-resolved measurements of the diffracted light from the multilayer structure. (**b**) The corresponding band gap showing an equivalent photonic structure of ∼28 bilayers. (**c**) An optical microscope image of the slanted cross-section of the multilayer grating.

We recorded multilayer structures by titling the sample at different angles with respect to the standing wave. The fabricated gratings corresponded to the slanted cross-section of a multilayer structure. We illuminated the Bragg grating at normal incidence with a supercontinuum white light laser. The backscatter light was measured at different angles using a spectrophotometer positioned onto a goniometer (Fig. 3a). The band gap of the photonic structure had a peak at ∼527 nm and a full width at half maximum bandwidth Δ*λ* of 36 nm. The 5-nm blue shift with respect to the recording laser was consistent in different trials. The blue shift could be attributed to a temperature difference during recording and readouts. During recording, the composite slightly expanded because of heat, whereas during readout the sample was at room temperature. An approximation of the recorded multilayer length can be extracted by considering a ‘weak-grating' approximation 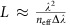 (‘Refractive index measurement' in Methods). The resulting length of the multilayer Bragg grating is ∼5.39 μm corresponding to ∼29 bilayers (Fig. 3b). In addition, the modulation in the effective refractive index Δ*n*_eff_ through the expression is 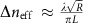 for a given diffraction efficiency of *R*. The estimated change in refractive index between fringes 2Δ*n*_eff_ is ∼0.01. The fabricated nanostructure served as a narrow-band wavelength-selective filter to diffract an intense colour at 8° away from the sample normal. Figure 3c shows an optical microscope image of a Ag NP transmission grating in the pHEMA matrix, consisting of a sinusoidal pattern (∼3 μm).

### Rewritable nanophotonic devices

**Figure 4:**
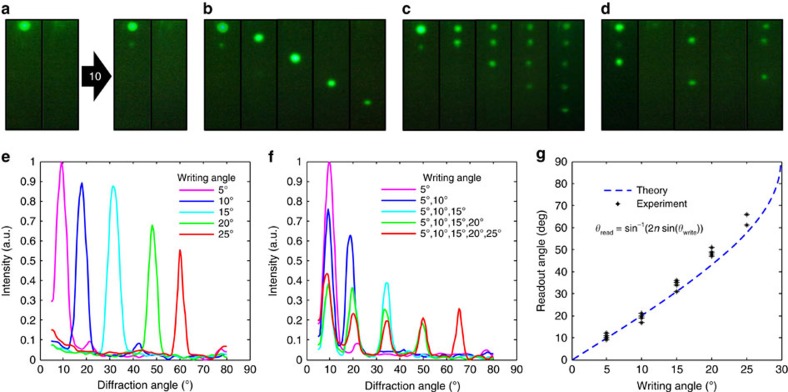
Assembly of reversible nanophotonic structures. (**a**) Multilayer structure at 5° of tilt angle after ten write–erase cycles. (**b**) Photonic crystal recorded and erased in Denisyuk reflection mode at 5°, 10°, 15°, 20° and 25°. (**c**) Assembly of a multiplexed photonic structure with the superposition of different crystal planes in a sample. (**d**) Dynamic binary information storage in a crystal structure showing reversibility. (**e**,**f**) Angle-resolved intensity measurements observed by the sample in **b** and **c**, respectively. (**g**) Theoretical and experimental readout angles from different Denisyuk mode writing angles.

To demonstrate reversibility, we recorded a grating at 5° from the surface plane and erased it (recorded at 0°) iteratively for 12 times (Fig. 4a, Supplementary Note 4 and Supplementary Fig. 3). The holographic patterning technique can be used to configure different crystal plane orientations (Supplementary Note 5 and Supplementary Fig. 4). Bragg planes were superposed at 5°, 10°, 15°, 20° and 25° to form photonic crystals (Fig. 4b). To erase the pattern, pHEMA matrix was aligned parallel to the surface plane of the object (that is, front-surface mirror) at 0°. This configuration aligned the multilayer structure with the specular reflection (zero order). Figure 4c shows the diffraction of the five band gaps projected at a semitransparent screen. Crystal structures in binary configurations were also recorded and erased, to demonstrate volumetric data storage (Fig. 4d). The angle-resolved diffraction intensity of the crystal structures were measured with a 532-nm laser (Fig. 4e,f corresponding to the structures in Fig. 4b,c, respectively). The diffracted rays diverged for large angles, because the grating structures embedded in pHEMA matrix had a high refractive index (∼1.43). Figure 4g shows a comparison of the different recorded angles and their theoretical predictions. The diffraction efficiency of the photonic structure decreased as the number of superposed spots increased. When NPs were distributed in multiple fringe planes, the contrast of the effective refractive index decreased.

**Figure 5:**
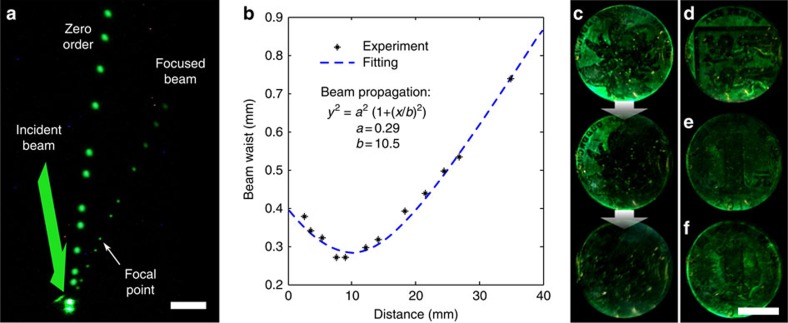
Active optical elements and 3D holograms. (**a**) A reconfigurable lens with a focal distance of 10 mm reconstructed with multiple images of the beam path (scale bar, 5 mm). (**b**) Beam waist measured along the path corresponding to the reconfigurable lens. (**c**) A 3D hologram of a coin recorded with interference of beams and its erasing process (scale bar, 1 cm). (**d**,**e**) Two different coins recorded after an erasing process. (**f**) Two coins superposed without an intermediate erasing process.

We also used the optical forces to fabricate dynamic lenses. A concave mirror with a 10-mm focal point was recorded. The focusing of a beam produced by the holographic lens is demonstrated with the superposition of multiple images of the beam path (Fig. 5a). Figure 5b shows a plot of the beam waist through the optical path. In addition, we obtained 3D holographic reconstructions of coins using multiple pulses at 10 Hz by recording a single object within 5 s. On white light illumination, a 3D holographic reconstruction was observed by the eye. The 3D reconstruction exhibited uniform monochromatic brightness. The holographic pattern was completely erased using the same laser beam within 30 s (Fig. 5c). Figure 5d,e shows different coins recorded within the same media after an erasing step. We also demonstrate image multiplexing capability by superposing two coins (Fig. 5f). By changing the number of pulses and intensity, the object beam can be finely tuned to partially or fully erase a 3D virtual image, or superpose a new hologram.

## Discussion

To date, the theoretical and empirical demonstrations of optical forces on dielectric or plasmonic particles have been mostly restricted to optical tweezers for the manipulation of NPs in liquids. We presented a mechanism to assemble volumetric nanostructures with optical forces. We showed that NPs could be organized inside solid matrixes, where NPs migrate to lower-energy configurations. Through this mechanism, nanomaterials can be reversibly assembled in stable configurations. The optical fluence necessary to observe this phenomenon with a nanosecond pulsed laser is 1–10 mJ cm^−2^. The presented strategy allows fabricating nanocomposite materials that have many attractive characteristics including fast and large area recording and reconfigurability. Reconfigurable composites can be combined with optical multiplexing techniques to store multiple holograms or volumetric data. In addition, lenses and virtual 3D reconstructions can be dynamically recorded. This mechanism is size scalable and it holds promise for a new generation of adaptive optical elements, displays, biosensors, data storage devices and metamaterials.

## Methods

### Fabrication of the recording media

3-(Trimethoxysilyl)propyl methacrylate in acetone (1:50, v/v) was poured onto glass microscope slides in an aluminum tray. After thorough coating, the excessive silane was poured off, while slides remaining *in situ* due to surface tension. The slides were stored in the tray overnight in the dark, before removal and dark storage at 24 °C. A monomer solution consisting of 2-hydroxyethyl methacrylate (91.5 mol%), ethylene dimethacrylate (2.5 mol%) and methacrylic acid (6 mol%) was prepared. The monomer solution was mixed with 1:1 (v/v) with photoinitiator 2,2-dimethoxy-2-phenylacetophenone in propan-2-ol (2%, w/v). A few silica beads (∼10 μm) were added to the solution. The monomer solution (200 μl) was deposited on an aluminized sheet and the glass slide, silanized treated side down, was placed on the solution to allow the capillary force to spread the solution throughout the surface the glass slide, whereas the silica beads controlled the thickness of the monomer solution. The monomer mixture was cured using ultraviolet light-induced free radical polymerization for 1 h. The pHEMA matrix was rinsed with ethanol (100 vol%) to remove the unreacted compounds from the pHEMA matrix. AgNO_3_ solution (0.1 M, 200 μl) dissolved in deionized water (DI) was perfused into the pHEMA matrix for 1 min. The excess salt solution was removed with a squeegee and the film was dried under tepid air current for 5 s. With the pHEMA matrix side facing up, the slide was submerged in a Petri dish containing LiBr (0.3 M, 40 ml) in methanol:water (3:2, v/v) for 30 s to convert the Ag^+^ ions to AgBr nanocrystals (∼10–30 nm). The slide was washed thoroughly with DI water to remove the unreacted Ag^+^ ions from the pHEMA matrix. The pHEMA matrix was exposed to broadband white light to desensitize the AgBr nanocrystals. A photographic developer (JD-4) consisting of 4-methylaminophenol sulfate (0.3%, w/v), ascorbic acid (2%, w/v), Na_2_CO_3_ (5%, w/v) and NaOH (1.5%, w/v) was prepared. The pHEMA matrix was immersed into the JD-4 developer (pH∼13) for 30 s, to allow the reduction of AgBr nanocrystals to metal Ag^0^ NPs. The pHEMA matrix was washed thoroughly with DI water and immersed into a stop bath consisting of acetic acid (5%, v/v, pH∼2.5), to neutralize the developer, for 10 s. The pHEMA matrix was submerged in a Na_2_S_2_O_3_ (10%, w/v) (hypo/X-ray fixer) solution to remove the unreacted AgBr nanocrystals. The images were recorded in the pHEMA matrix in ambient humidity (60% relative humidity (RH)).

### Recording in Denisyuk reflection mode

**Figure 6:**
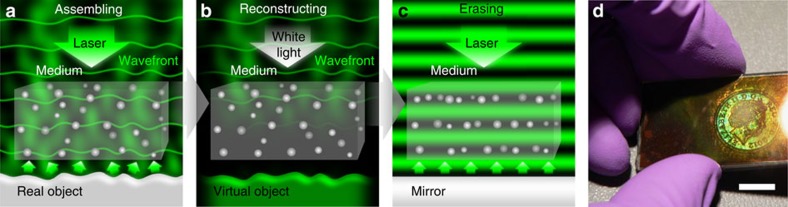
Holographic assembly in Denisyuk reflection mode. (**a**) The interference pattern produced by a reference beam in the wave front of an object creates an assembly of NPs in the medium. (**b**) The wavefront is reconstructed with a broadband light source. (**c**) The reconstruction is erased by arranging the NPs in a multi-layer plane parallel to the surface. (**d**) Recorded sample with Ag NPs embedded in a pHEMA medium on a glass substrate (scale bar, 1 cm).

The standing waves were formed via in-line Denisyuk reflection mode^41^. Figure 6 illustrates the experimental setup schematic. Laser pulses were directed onto a pHEMA recording medium and reflected from the recording object, which was placed over a translational stage and positioned normal to the laser incidence pulse having a spot size of ∼1 cm in diameter (Fig. 6a). The semi-transparent composite is placed between the reference beam and the object reflection beam. In the case of the volume grating, the medium was tilted an angle from the surface mirror plane. The laser pulse energy at the sample–air interface was measured as ∼10 mJ, using an optical powermeter. The incident pulse propagated through the substrate and the pHEMA recording medium, and reflected off from an object. The interference of the object beam and the reference beam created a standing wave. The recorded object was reconstructed with white light without affecting the assembly (Fig. 6b). The assembly was arranged in multi-layer planes parallel to the surface interface to erase the reconstruction (Fig. 6c). Figure 6d shows a 3D reconstruction observed from a sample recorded in Denisyuk reflection mode.

### Refractive index measurement

The refractive index of the pHEMA was measured by an Abbé refractometer. The pHEMA matrices were lifted off from their substrates, lubricated with an index matching fluid and placed on the reading plate of the refractometer. The measured refractive index of pHEMA matrix without NPs was 1.37. However, this value increased to 1.43 once the NPs were added into the pHEMA matrix. We used an analytical solution for the non-absorbing effective refractive index oscillation Δ*n* of an embedded grating^42^:


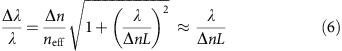


and


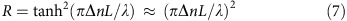


Through this method we obtained a value Δ*n* of ∼0.005. The light absorption was not considered in these equations.

### Transmission electron microscopy imaging

pHEMA matrix was released from its glass substrate with a razor blade and transferred to dry ethanol. This solution replaced with two changes of CH_3_CN for 10 min each. The pHEMA matrix was transferred into a mixture (50:50, v/v) of CH_3_CN and Quetol 651. The CH_3_CN was allowed to evaporate for 12 h. The pHEMA matrix was transferred through three changes of Quetol 651 for 2 h each and the resin was cured at 60 °C for 48 h. Vertical sections through the pHEMA matrix were cut with a diamond knife using a microtome. The samples were mounted on Cu grids (400 mesh) and viewed in a transmission electron microscope (TEM) operating at 120 kv. TEM images were obtained on a FEI Tecnai system (Oregon, USA). The TEM images were recorded with an Advanced Microscopy Techniques (AMT) 60B camera running Deben image acquisition software.

### Materials and instruments

3-(Trimethoxysilyl)propyl methacrylate (98%), 2-hydroxyethyl methacrylate (99+%), ethylene dimethacrylate (98%), methacrylic acid (99%), 2, 2-dimethoxy-2-phenylacetophenone (99%), AgNO3 (99%), L-ascorbic acid (99%), Na_2_CO_3_ (99.9%) and NaOH (98.0%) were purchased from Sigma-Aldrich. 4-Methylaminophenol sulfate (metol) (99%) was purchased from Acros Organics. Microscope slides (1.2 mm thick) were purchased from Fisher Scientific. Single-side aluminized polyester film was purchased from HiFi Industrial Film Ltd (Stevenage, UK). Stratalinker 2,400 UV Crosslinker (∼350 nm, 4,000 μW cm^−2^) was purchased from RS Components (Corby, UK). Nd-Yttrium-Aluminum-Garnet pulsed laser (high-power compact Q-switched Nd:YAG oscillator with super Gaussian resonator, 700 mJ at 1,064 nm, 10 Hz) with a second harmonic generator, 350 mJ at 532 nm and 10 Hz, thermally stabilized with wavelength separation) and supercontinuum white light laser were purchased from Lambda Photometrics (Harpenden, UK) and Fianium (SC400, Eugene, OR), respectively. AvaSpec 2,028 spectrophotometer, 2,048-pixel InstaSpec IV charge-coupled device detector, QE6500 spectrometer and a bifurcated cable (FC UV 600–2, 600 μm fibre, 2 m length, SMA terminations) were purchased from Avantes (Apeldoorn, The Netherlands) and Ocean Optics (Dunedin, FL). Abbé refractometer (Atago 4t) was purchased from Atago USA (Bellevue, WA). A plano-convex lens (laser grade PCX lens, 25 mm diameter × 75 mm FL, uncoated) was purchased from Edmund Optics Ltd (York, UK). AvaSoft (v7.5) software, Igor Pro, COMSOL Multiphysics (v5.1) and MATLAB (MathWorks, v8.1) were used for data processing and finite element simulations. dEYEmond HISTO 5/6/8-mm microtome diamond knife was purchased from Scimed GmbH (Am Mühlenkanal, Germany) and a low-temperature sectioning system (microtome) Ultracut UCT was purchased from Leica (Vienna, Austria). NP size distributions were analysed using ImageJ (v1.48p, 64 bit, National Institutes of Health, USA).

### Data availability

The data that support the findings of this study are available from the corresponding author upon request.

## Additional information

**How to cite this article:** Montelongo, Y. *et al*. Reconfigurable optical assembly of nanostructures. *Nat. Commun.* 7:12002 doi: 10.1038/ncomms12002 (2016).

## Supplementary Material

Supplementary Information Supplementary Figures 1-4, Supplementary Notes 1-5 and Supplementary References.

Supplementary Movie 1 Dynamic schematic of the holographic nanoassembly of a photonic crystal. In the process, metallic NPs absorb light increasing the temperature of the surrounding medium. The reduction in viscoelasitic at the boundaries of the NPs allow them to migrate to lower-energy configurations. After the thermal energy dissipates, NPs remain in steady positions.
