# Supplementary material for: Reconfigurable optical assembly of nanostructures
Source: Nat Commun. 2016 Jun 23;7:12002. doi: 10.1038/ncomms12002 (PMC4931027; doi:10.1038/ncomms12002)
Supplement: Supplementary Information — Supplementary Figures 1-4, Supplementary Notes 1-5 and Supplementary References. [file ncomms12002-s1.pdf]

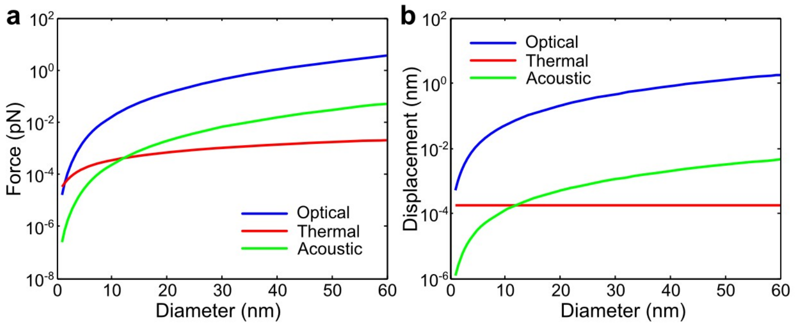

Supplementary Figure 1 Different types of migrations. Order of magnitude of the **a** force and **b** displacement achieved by optical, thermal and acoustic migrations of Ag NPs in a pHEMA matrix.

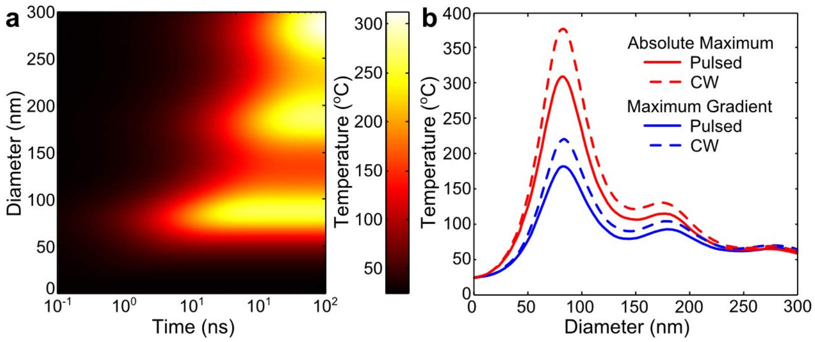

Supplementary Figure 2 Temperature at the NP-pHEMA boundary. a. Absolute maximum temperature reached in Ag NPs from a CW laser at 532 nm. **b.** Temperature observed after 5 ns from the CW model compared with the pulsed model (Fig. 1c) for NPs at the maximum intensity and maximum gradient point in the standing wave.

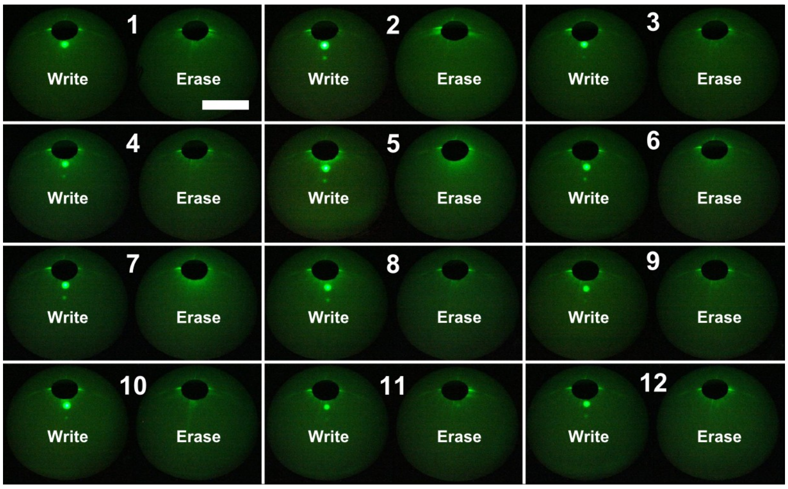

Supplementary Figure 3 Rewritable Bragg grating. Write-erase cycles repeated 12 times (scale bar 1 = cm).

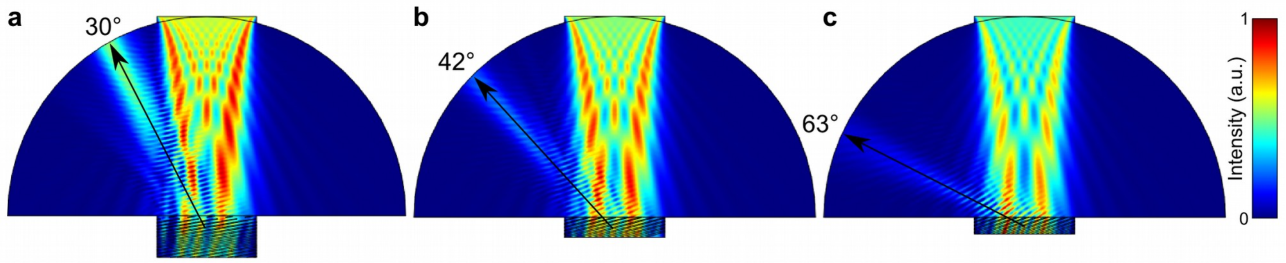

Supplementary Figure 4 Computation of the interaction between the nanostructure and the electromagnetic field. Three different structures were retrieved at **a** 15°,**b** 20° and **c** 25° in Denisyuk mode producing diffraction at 30°, 42° and 63° respectively.

Supplementary Note 1: Migration by optical forces

To find the displacement of the NP, the shear modulus *G* of the medium should be defined. In the case of polymers, there is a frequency and temperature dependence. Furthermore, porous materials can change their modulus by several orders of magnitude in humid conditions. A common technique to retrieve the viscoelastic behavior of solids is by measuring the spectral response. The shear modulus $G(\omega)=G'(\omega)+iG''(\omega)$ is a complex number, where the real part represents the conservative elasticity (storage modulus), and the imaginary the non-conservative parameter (loss modulus). The real and the imaginary parts are represented as a function of the angular frequency ω.

In a typical polymer, the complex shear modulus tends to be constant in two frequency regimes: the rubbery regime (low shear frequencies) and the glassy regime (high shear frequencies). Additionally, between these two regimes, there is a transition regime where the storage and loss modulus change exponentially. According to experiments performed by Lustig and Meakin[1,2] in pHEMA, temperature and water content produce an analogous mechanical frequency shift. This implies that when temperature and water content increases, high frequencies behave as low frequencies. The translation of frequencies permits the polymer to behave as rubber and not as glass even at high frequencies. The rubbery complex storage modulus of pHEMA is $G=2.9\times10^4+i2.0\times10^4$ Pa, and the glassy complex storage modulus is $G=1.4\times10^9+i2.0\times10^7$ Pa[1,2]. At relative humidity (RH) of 60% and 24 C°, pHEMA is rubbery with a constant shear modulus in the frequency regime of interest. This approximation is in agreement with Lustig and Meakin's measurements and our experimental data.

Stokes described the relation between the speed and the force of a sphere embedded in a fluid with constant viscosity. However, Stokes' law cannot be applied for media with frequency dependent

viscoelasticity (*e.g.* solids). The generalized Stokes' equation describes the displacement produced by a sphere in a solid with a shear modulus $G(\omega)$ and an oscillating force $F_\omega(\omega)$[3]:

$$z_\omega(\omega)=\frac{F_\omega(\omega)}{6\pi r G(\omega)} \quad 1$$

The complex frequency dependent position $z_\omega(\omega)$ provides the amplitude and phase components of the oscillation. Both, the real and imaginary parts of the shear modulus contribute to the displacement of the NP. However, only the non-conservative part $iG''(\omega)$ produces permanent migration.

To obtain the displacement, we define the average intensity of a standing wave produced by a Gaussian pulse of energy U_p and width σ (the standard deviation σ of the Gaussian can be obtained with the Full Width at Half Maximum (FWHM) t_p with the relation $t_p=\sigma 2\sqrt{2\ln(2)}$). In the case of two counter propagating waves normal to the z axis with phase zero, and wave number $k=2\pi\frac{n_{\mathrm{eff}}}{\lambda}$, the average intensity profile is:

$$I(z,t)=2(1+\sin(2kz))\frac{U_p}{\sigma\sqrt{2\pi}}e^{\frac{-t^2}{2\sigma^2}} \quad 2$$

The component of the gradient $\mathbf{\nabla}I$ in the direction of propagation is:

$$\frac{dI}{dz}=4k\cos(2kz)\frac{U_p}{\sigma\sqrt{2\pi}}e^{\frac{-t^2}{2\sigma^2}} \quad 3$$

The optical force is proportional to the gradient of the irradiance $\mathbf{F}_p \propto \nabla I$. A relative maximum force F_p^{max} is defined as a proportionality constant that satisfies:

$$\mathbf{F}_p=F_p^{max}\nabla I \quad 4$$

The value of the force in the z axis becomes:

$$F_{\mathrm{p}} = 4 F_{\mathrm{p}}^{\max} k \cos(2kz) \frac{U_p}{\sigma\sqrt{2\pi}} e^{\frac{-t^2}{2\sigma^2}} \quad 5$$

and in frequency domain (Fourier transform) is:

$$F_{\omega}(\omega) = 4 F_{\mathrm{p}}^{\max} k \cos(2kz) U_{\mathrm{p}} e^{\frac{-\sigma^2\omega^2}{2}} \quad 6$$

The term $\cos(2kz)$ is considered constant because the displacement during the pulse is minimal compared to the wavelength. Hence, we find the frequency dependent displacement.

$$z_{\omega}(\omega) = \frac{2 F_{\mathrm{p}}^{\max} k \cos(2kz) U_{\mathrm{p}}}{3\pi r} \frac{e^{\frac{-\sigma^2\omega^2}{2}}}{G(\omega)} \quad 7$$

The total amplitude is obtained with the contribution of all frequencies:

$$z_{\mathrm{p}} = \frac{2 F_{\mathrm{p}}^{\max} k \cos(2kz) U_{\mathrm{p}}}{3\pi r} \left| \int_{-\infty}^{\infty} \frac{1}{G(\omega)} e^{\frac{-\sigma^2\omega^2}{2}} d\omega \right| \quad 8$$

The non-conservative displacement is found with the imaginary part of the shear modulus $iG''(\omega)$. In contrast to fluids, solids tend to have constant shear modulus in large regimes. When $G''(\omega)$ is approximated as a constant, the total displacement can be obtained as:

$$z_{\mathrm{p}} = \frac{2 F_{\mathrm{p}}^{\max} k \cos(2kz) U_{\mathrm{p}}}{3\pi r G''} \left(\frac{\sqrt{2\pi}}{\sigma} \right) \approx 1.25 \frac{F_{\mathrm{p}}^{\max} k \cos(2kz) U_{\mathrm{p}}}{r G'' t_{\mathrm{p}}} \quad 9$$

For high repetition rates, the total displacement with differentials is:

$$dz = 1.25 \frac{F_{\mathrm{p}}^{\max} k \cos(2kz)}{r G'' t_{\mathrm{p}}} dU \quad 10$$

Starting from z_0 to a position z:

$$\int_{z_0}^{z} \frac{1}{\cos(2kz)} dz = \int_0^U 1.25 \frac{F_p^{max} k}{r G'' t_p} dU \quad 11$$

Then:

$$\ln|\tan(kz+\pi/4)| - \ln|\tan(kz_0+\pi/4)| = 2.5 \frac{F_p^{max} k^2}{r G'' t_p} U \quad 12$$

The displacement from the maximum gradient point (z_0=0) after a total exposure energy U is:

$$\Delta z = \arctan(\exp(\frac{2.5 F_p^{max} k^2}{r G'' t_p} U))/k - \frac{\pi}{4k} \quad 13$$

which can be approximated close to maximum gradient point as:

$$\Delta z \approx \frac{1.25 F_p^{max} k}{r G'' t_p} U \quad 14$$

Supplementary Note 2: Thermophoretic and acoustophoretic migration

Thermal and acoustic forces occur due to gradients of temperature in the medium. In thermophoresis (or thermodiffusion), the entropy of NPs results in migration from high to low intensity regions. In acoustophoresis, a transient of temperature produces a mechanical pressure that migrates NPs to low intensity regions. These two properties can be applied to model the temperature distribution, either conceptualizing the composite as homogeneous or inhomogeneous. Since NPs are randomly arranged in the pHEMA matrix, we approximate the composite as homogeneous to retrieve the effective behavior.

Heat and pressure can be highly dissipative at the nanoscale[4]. Considering the thermal diffusivity of the medium α_m, the pulse length should be shorter than the confinement of temperature

$t_p < \frac{\lambda^2}{32 n_m^2 \alpha_m}$. If the heat source is constant, the temperature gradient can also be approximated as a constant after this period. Pressure dissipation occurs at higher speed than heat because acoustic

waves propagate faster. Considering the speed of sound in the medium c_m, the pulse length should be shorter than the acoustic confinement $t_p < \frac{\lambda}{4 n_m c_m}$. In the present work, the thermal confinement is about three fold larger than the pulse length, and therefore, we approximate the energy transfer from light as instantaneous. However, pressure confinement is about two orders of magnitude shorter than the pulse length. Hence, the effective difference of pressure between fringes is considered.

In thermophoresis, the force $\mathbf{F}_T$ produced is proportional to the gradient of temperature $\mathbf{F}_T \propto \nabla T$ [5]. The thermodiffusive velocity of the NP is:

$$\frac{d\mathbf{x}}{dt} = -D_c \nabla T \quad 15$$

where D_c is the thermodiffusion coefficient of the composite. Although D_c is well established in aerosols, its value remains controversial for liquids and solids[6,7]. In the present work we applied Brenner's model, which has shown consistency with the experimental work for viscous liquids[5,8]:

$$D_c = \frac{\alpha_m , \beta_m}{1 + (K_{NP}/(2K_m))} \quad 16$$

where α_m, β_m, K_m, are the thermal diffusivity, thermal expansion and thermal conductivity of the medium respectively, and α_m and K_{NP} are the thermal diffusivity and thermal conductivity of the NP.

The heat equation can describe the distribution of temperature in time. When the pulse time is shorter as compared to the heat diffusion time, the optical energy U_p is transferred instantaneously to thermal energy U_t producing an increment of temperature ΔT. For a standing wave at time zero ΔT is:

$$\Delta T = \frac{\alpha_{eff}}{K_{eff}} U_t = \frac{\alpha_{eff}}{K_{eff}} \sigma \int_{-\infty}^{\infty} 2 I_0 (1 + \sin(2kz)) \, dt = 2\sigma U_p \frac{\alpha_{eff}}{K_{eff}} (1 + \sin(2kz)) \quad 17$$

where α_{eff} and K_{eff} are the effective diffusivity and conductivity of the composite, and σ is the optical absorption coefficient from Beer's law (the properties of the medium can be used for low

concentration of NPs)[9]. After the optical energy produces an increment in the temperature, the heat diffuses with time until the gradient disappears. Based on the heat equation, a sinusoidal temperature function remains sinusoidal while it dissipates heat but decreases in its intensity as $\exp(-4\alpha_{\mathrm{m}} k^2 t)$. The component of the gradient $\boldsymbol{\nabla} T$ in the direction of propagation with the exponential decrement in time is:

$$\frac{dT}{dz}=4k\sigma U_{\mathrm{p}}\frac{\alpha_{\mathrm{m}}}{K_{\mathrm{m}}}\cos(2kz)e^{-4\alpha_{\mathrm{m}}k^2 t} \quad 18$$

By combining 18 and 15, the thermophoretic velocity is:

$$\frac{dz}{dt}=-4kD_{\mathrm{c}}\sigma U_{\mathrm{p}}\frac{\alpha_{\mathrm{m}}}{K_{\mathrm{m}}}\cos(2kz)e^{-4\alpha_{\mathrm{m}}k^2 t} \quad 19$$

and the total thermophoretic displacement Δz_{T} from the maximum gradient point is:

$$\Delta z_{\mathrm{T}}=\operatorname{arccot}(\exp(\frac{2D_{\mathrm{c}}\sigma U_{\mathrm{p}}}{K_{\mathrm{m}}}))/k-\frac{\pi}{4k}\approx-\frac{D_{\mathrm{c}}\sigma U_{\mathrm{p}}}{kK_{\mathrm{m}}} \quad 20$$

The thermodiffusive force $\mathbf{F}_{\mathrm{T}}$ at the maximum gradient point is approximated with the generalized Stokes' law:

$$\mathbf{F}_{\mathrm{T}}\approx-\frac{6\pi rG''D_{\mathrm{c}}\sigma U_{\mathrm{p}}}{kK_{\mathrm{m}}} \quad 21$$

Additionally, acoustic forces arise when a difference in pressure is produced in the medium due the increment of temperature. By analyzing the fluid dynamics of a rigid sphere in a liquid, the acoustic force exerted $\mathbf{F}_{\mathrm{a}}$ and the acoustic energy U_{a} is correlated[10]:

$$\mathbf{F}_{\mathrm{a}}=-2\pi r^3\boldsymbol{\nabla} U_{\mathrm{a}} \quad 22$$

If the acoustic energy is expressed in terms of the pressure difference Δp and the density of the material ρ_{m}, the acoustic force is:

$$\mathbf{F}_{\mathrm{a}}=\frac{-\pi r^3}{\rho_{\mathrm{m}} c_{\mathrm{m}}^2}\nabla(\Delta p)^2 \quad 23$$

The pressure difference can be found with the solution of the inhomogeneous wave equation, where the input source is extracted from the difference in temperature. To simplify, the difference in pressure is considered constant. Its value is approximated with the difference in temperature achieved during the confinement time (the effective reduction is proportional to $\frac{\lambda}{4 n_{\mathrm{m}} c_{\mathrm{m}} t_{\mathrm{p}}}$ [4]):

$$\Delta p \approx \beta_{\mathrm{m}} \rho_{\mathrm{m}} c_{\mathrm{m}}^2 \Delta T\left(\frac{\lambda}{4 n_{\mathrm{m}} c_{\mathrm{m}} t_{\mathrm{p}}}\right)=\frac{\lambda \beta_{\mathrm{m}} \rho_{\mathrm{m}} c_{\mathrm{m}} \sigma \alpha_{\mathrm{m}} U_{\mathrm{p}}}{2 n_{\mathrm{m}} t_{\mathrm{p}} K_{\mathrm{m}}}(1+\sin(2 k z)) \quad 24$$

Hence, the acoustic force as a function of position is:

$$F_{\mathrm{a}}=-\frac{8\pi^3 r^3 \rho_{\mathrm{m}}}{k}\left(\frac{\sigma \beta_{\mathrm{m}} \alpha_{\mathrm{m}} U_{\mathrm{p}}}{K_{\mathrm{m}} t_{\mathrm{p}}}\right)^2 \cos(2 k z)(1+\sin(2 k z)) \quad 25$$

Similarly, the displacement is extracted from the generalized Stokes' law:

$$\Delta z_{\mathrm{a}}=\frac{4\pi^2 r^2 \rho_{\mathrm{m}}}{3 G'' k}\left(\frac{\sigma \beta_{\mathrm{m}} \alpha_{\mathrm{m}} U_{\mathrm{p}}}{K_{\mathrm{m}} t_{\mathrm{p}}}\right)^2 \cos(2 k z)(1+\sin(2 k z)) \quad 26$$

and the total displacement at the maximum gradient point Δz_{a} after a pulse of length t_{p} is approximated as:

$$\Delta z_{\mathrm{a}} \approx \frac{4\pi^2 r^2 \rho_{\mathrm{m}}}{3 G'' k}\left(\frac{\sigma \beta_{\mathrm{m}} \alpha_{\mathrm{m}} U_{\mathrm{p}}}{K_{\mathrm{m}} t_{\mathrm{p}}}\right)^2 \quad 27$$

Both, acoustophoretic and thermophoretic models are punctual. For NPs of size comparable to the wavelength it is necessary to consider the wrapping of the standing wave. Supplementary Figure 2a and 2b shows the order of magnitudes of the force and displacement respectively at the maximum gradient point achieved by optical (blue), thermal (red) and acoustic (green) foreces for Ag NPs embedded in pHEMA medium with a pulsed Nd:YAG laser (532 nm and 5 ns). It can be concluded that, under these conditions, NPs migrate predominantly by optical forces.

Supplementary Note 3: Heating model

The temperature of the system can be obtained by solving the heat equation of a sphere embedded in the pHEMA matrix. In the case of radiation I_{CW}, the solution of the heat equation in time and space for a sphere of radius r_s can be approximated as:

$$T_{CW}(r,t)=I_{CW}\,q\left(1-e^{-\frac{\alpha_{NP}}{K_{NP}T_{\infty}(r)}t}\right)T_{\infty}(r) \quad 28$$

where K_{NP}, α_{NP} are the thermal conductivity and diffusivity of the NP respectively. The variable q is the heat per unit of volume per watt when a NP absorbs light:

$$q=\frac{3}{4}\frac{C_{ab}}{r_s} \quad 29$$

The absorption efficiency C_{ab} is retrieved from the GLMT (0.1-0.3 for Ag NPs in this regime). The function $T_{\infty}(r)$ represents the equilibrium temperature that is achieved after an infinite time:

$$T_{\infty}(r)=\begin{cases}\left(\frac{r_s^2}{3K_m}+\frac{r_s^2-r^2}{6K_{NP}}\right) & for\ \ r<r_s\ \ (inside)\\ \left(\frac{r_s^3}{3rK_m}\right) & for\ \ r>r_s\ \ (outside)\\ \left(\frac{r_s^2}{3K_m}\right) & for\ \ r=r_s\ \ (surface)\end{cases} \quad 30$$

The time dependence of the temperature at the surface of the NP is reduced to:

$$T_{CW}(r_s,t)=I_{CW}C_{ab}\left(\frac{r_s}{4K_m}\right)\left(1-e^{-\beta t}\right) \quad 31$$

where

$$\beta=\frac{3K_m\alpha_{NP}}{K_{NP}r_s^2} \quad 32$$

Supplementary Figure 1a shows the temperature at the surfaces of Ag NPs embedded in pHEMA for a continuous wave (CW) laser with an intensity I_{CW} = 40 GW m^{-2} until equilibrium (as analogy of the intensity found at the maximum gradient point for two counter-propagating pulses of 10 mJ cm^{-2} in 5 ns). Similarly, the solution for a Gaussian pulse has been calculated numerically according to the experimental parameters. Supplementary Figure 1b illustrates the results for the absolute maximum of temperatures in different NP diameters and at the maximum gradient point for CW and a Gaussian laser pulse.

Supplementary Note 4: Reconfigurability

The recorded pattern can be erased by arranging a multilayer structure parallel to the mirror surface plane. This process aligns the diffraction with the specular reflection (zero order). The previous pattern can be partially erased or fully erased depending on the number of pulses applied. The patterning process is reversible and the same emulsion may be used for multiple times without any noticeable change in diffraction efficiency. We have repeated this process over 100 times by using different objects with no fatigue in the composite (Supplementary Figure 3).

Supplementary Note 5: Photonic structure model

Finite element method (FEM) was used for simulating the diffraction behavior of the Bragg gratings recorded at different angles. A MATLAB code was used for calculating the standing wave interference patterns (between emitted and reflected beams in Denisyuk mode) within the pHEMA matrix, when illuminated at different angles. These interference patterns are eventually responsible for configuring the 3D arrangement of NPs within the pHEMA matrix, producing the optical structures. Additionally, the internal reflection was considered in the model. The interference patterns were imported into the FEM code and treated as effective refractive index regions with and without NPs. The effective refractive index used was 1.42 with an oscillation Δn of 0.01. In the model, a broadband light source was irradiated on the photonic Bragg gratings and the resulting reflection and diffraction properties were analyzed. The gratings reflected broadband light normally and diffracted monochromatic light at angles approximately twice the angle used for holographic writing (Supplementary Figure 4).

Safety Considerations for Laser Use: The fabrication method described involves the use of a class IV laser. The operators must be trained and aware of the hazards of laser exposure to the bare skin and eye. Appropriate laser wavelength rated safety googles must be worn and the lasers must be operated with safety precautions. For example, reflection of laser light from mirrors and other reflecting surfaces described in the experimental section may cause damage to an untrained researcher.

Supplementary References

1. Lustig, S. R., Caruthers, J. M. & Peppas, N. A. Dynamic mechanical properties of polymer-fluid systems: characterization of poly(2-hydroxyethyl methacrylate) and poly(2-hydroxyethyl methacrylate-co-methyl methacrylate) hydrogels. *Polymer* **32,** 3340–3353 (1991).
2. Meakin, J. R., Hukins, D. W. L., Aspden, R. M. & Imrie, C. T. Rheological properties of poly(2-hydroxyethyl methacrylate) (pHEMA) as a function of water content and deformation frequency. *J. Mater. Sci. Mater. Med.* **14,** 783–787 (2003).
3. Gittes, F., Schnurr, B., Olmsted, P. D., MacKintosh, F. C. & Schmidt, C. F. Microscopic Viscoelasticity: Shear Moduli of Soft Materials Determined from Thermal Fluctuations. *Phys. Rev. Lett.* **79,** 3286–3289 (1997).
4. Wang, L. V. Tutorial on Photoacoustic Microscopy and Computed Tomography. *IEEE J. Sel. Top. Quantum Electron.* **14,** 171–179 (2008).
5. Schermer, R. T., Olson, C. C., Coleman, J. P. & Bucholtz, F. Laser-induced thermophoresis of individual particles in a viscous liquid. *Opt. Express* **19,** 10571 (2011).
6. Duhr, S. & Braun, D. Why molecules move along a temperature gradient. *Proc. Natl. Acad. Sci.* **103,** 19678–19682 (2006).
7. Young, J. B. Thermophoresis of a Spherical Particle: Reassessment, Clarification, and New Analysis. *Aerosol Sci. Technol.* **45,** 927–948 (2011).
8. Brenner, H. & Bielenberg, J. R. A continuum approach to phoretic motions: Thermophoresis. *Phys. Stat. Mech. Its Appl.* **355,** 251–273 (2005).
9. Cox, B., Laufer, J. G., Arridge, S. R. & Beard, P. C. Quantitative spectroscopic photoacoustic imaging: a review. *J. Biomed. Opt.* **17,** 0612021–06120222 (2012).
10. Dysthe, K. B. Force on a small inclusion in a standing acoustic wave. *J. Sound Vib.* **10,** 331–339 (1969).
